# COVID-19 Vaccine Hesitancy among Health Professional Students: Cross-Sectional Data from the First Wave of the HOLISTIC Cohort Study

**DOI:** 10.3390/vaccines10091566

**Published:** 2022-09-19

**Authors:** Daniel D. Loizzo, Avisek Datta, Sunil R. Dommaraju, Ummesalmah Abdulbaseer, Jerry A. Krishnan, Mary Keehn, Rashid Ahmed

**Affiliations:** 1 College of Medicine, University of Illinois Chicago, Chicago, IL 60612, USA; 2 School of Public Health, University of Illinois Chicago, Chicago, IL 60612, USA; 3 Population Health Sciences Program, Office of the Vice Chancellor for Health Affairs, University of Illinois Chicago, Chicago, IL 60612, USA; 4 College of Applied Health Sciences, University of Illinois Chicago, Chicago, IL 60612, USA; 5 Interprofessional Practice and Education, Office of the Vice Chancellor for Health Affairs, University of Illinois Chicago, Chicago, IL 60612, USA

**Keywords:** COVID-19, vaccine hesitancy, health professional students

## Abstract

Vaccine hesitancy has been observed around the world, but there is a paucity of data among a broad range of U.S. health professional students. The goal of this report is to present findings about COVID-19 vaccine hesitancy among a cross-section of U.S. health professional students and determine if hesitancy varies by demographic characteristics, health science college, and other factors. A cross-sectional analysis of HOLISTIC Cohort Study participants enrolled from April 14 2021 to May 5 2021 at seven health sciences colleges in the University of Illinois Chicago was used. Exploratory and confirmatory factor analysis were used to evaluate vaccine hesitancy items and identify domains. Among 555 health professional students, three domains (perceived benefit, trustworthiness, and risk) contribute to vaccine hesitancy. Significant differences were observed in the domains among students of different races as well as vaccination history. Compared to students in the College of Medicine, students in the Colleges of Applied Health Science (OR 0.43; CI [0.19–0.96]), Pharmacy (OR 0.38; CI [0.17–0.87]), Nursing (OR 0.35; CI [0.16–0.78]), and Social Work (OR 0.30; CI [0.11–0.78]) reported lower perceived benefit. Compared to students in the College of Medicine, students in the College of Applied Health Sciences (OR 0.39; CI [0.17–0.94]), Dentistry (OR 0.27; CI [0.10–0.76]), Nursing (OR 0.38; CI [0.16–0.94]), and Social work (OR 0.31; CI [0.11–0.86]) reported more trustworthiness and more concerns about risk (OR 2.80; CI [1.15–6.81] for College of Applied Health Sciences, OR 9.12; CI [2.80–29.75] for Dentistry, OR 3.77; CI [1.47–9.65] for Nursing, OR 3.14; CI [1.02–9.67] for Social Work). Our findings suggest the need for a tailored vaccination strategy among different subgroups of health professional students.

## 1. Introduction

As of July 2022, billions of doses of highly efficacious coronavirus disease 2019 (COVID-19) vaccines have been administered worldwide [1,2]. However, many individuals, particularly younger adults, are hesitant to be vaccinated against COVID-19 [3,4,5]. Various factors may contribute to COVID-19 vaccine hesitancy, including the potential for adverse effects [4,6,7,8], perceived risk of COVID-19 [5,8], trust [8,9,10,11], and disagreements about the ability of public health authorities to mandate vaccination [9,12].

Vaccine hesitancy has also been observed among health professionals [13,14]. Similar to observations in the general population, vaccine hesitancy is more common among health professionals who are younger [15] or have concerns about adverse effects [15,16,17], perceived risk of COVID-19 [15], inadequate efficacy [15,17,18], and inadequate trust [16]. A meta-analysis of studies of health professional students demonstrates that a similar proportion of trainees exhibit vaccine hesitancy compared to practicing healthcare professionals, and that students cite similar concerns about COVID-19 vaccination as practitioners [19]. Data about vaccine hesitancy among health professional students in the United States (U.S.) are largely limited to medical, dental, and nursing students [20,21,22,23]. There is a paucity of data about COVID-19 vaccine hesitancy among other U.S. health professional students, including kinesiology, nutrition, occupational and physical therapy, pharmacy, public health, and social work.

Vaccine uptake or refusal are behaviors that likely result from complex decision-making processes that could be influenced by a variety of factors [24]. In order to better understand the reasons a person may be more or less likely to receive a vaccine, the World Health Organization reviewed a number of theoretical frameworks in 2012 to characterize the factors associated with these behaviors [25,26]. One of the first proposed models was the “3Cs” model, which characterized vaccine hesitancy by three factors: complacency, convenience, and confidence [27]. Since the inception of the “3Cs”, multiple other models have been published and validated to explain the factors influencing vaccine hesitancy [27,28,29,30].

The objective of this report is to present findings about COVID-19 vaccine hesitancy among a broad cross-section of U.S. health professional students in April to May 2021, shortly after the emergency use authorizations for the first two COVID-19 vaccines in the U.S [31]. A secondary objective of this report is to determine if COVID-19 vaccine hesitancy among students varies by health science program and by demographic characteristics. The results of this study could inform the need for a tailored vaccination strategy among different subgroups of health professional students.

## 2. Materials and Methods

### 2.1. Study Design

The Health Professional Students at the University of Illinois Chicago (HOLISTIC) Cohort Study is a prospective cohort study with three waves of recruitment (Spring 2021, Spring 2022, Spring 2023) [32]. The current report is a cross-sectional analysis of baseline data from HOLISTIC Cohort Study participants enrolled in the first recruitment wave (14 April 2021 to 5 May 2021).

### 2.2. Study Population

The HOLISTIC Cohort Study enrolled students across seven health science programs (applied health sciences, dentistry, medicine, nursing, pharmacy, public health, and social work) at the University of Illinois Chicago, a U.S. Department of Education designated minority-serving institution [33]. Students were eligible to enroll in the HOLISTIC Cohort Study if they were age 18 years or older and enrolled full- or part-time in a health science program that prepares its graduates to enter a healthcare profession. Students were recruited via an email sent through their educational program’s listserv detailing the study and its eligibility requirements. More details about the design of the HOLISTIC Cohort Study are available in a previous publication [32].

### 2.3. Questionnaire

The HOLISTIC Cohort Study included two questionnaires, the U.S. Centers for Disease Control and Prevention’s (CDC) Behavioral Risk Factor Surveillance System (BRFSS) 2019 survey [34], and the 2014 World Health Organization Report of the Strategic Advisory Group of Experts (WHO SAGE) Working Group Vaccine Hesitancy Scale (VHS) [35]. The data on demographics and healthcare access presented in this report were based on items in the BRFSS survey. The specific questions analyzed from the BRFSS survey can be found in the study supplement (Table S1). The 10 items in the WHO SAGE VHS, each with five possible responses (strongly disagree, disagree, neutral, agree, and strongly agree) were adapted to assess hesitancy specific to COVID-19 vaccines. In the study supplement, we provide the original and modified wording (Table S2). An additional question was generated to assess desire to receive the COVID-19 vaccine, once available: (1) I would receive a vaccine developed for COVID-19 or coronavirus.

### 2.4. Statistical Analysis

Descriptive statistics were used to characterize the study population and survey responses. Exploratory and Confirmatory Factor Analysis (EFA and CFA) with varimax rotation was used to evaluate the vaccine hesitancy items and identify domains. Various domains, such as lack of confidence, risk factor concern, and misinformation, have been utilized in prior studies to analyze these questions [36,37]. The sample data was divided into two components, where half of the sample was used for EFA and the other half was used for CFA. EFA was performed using varimax rotation with eigenvalues > 1, enforcing a three-factor solution. Reliability analyses were used to assess the internal consistencies of each set of items. In the EFA, we performed maximum likelihood estimation and principal components analysis. Following EFA procedures, CFA was then performed using maximum likelihood estimation. Modeling goodness of fit measures such as Adjusted Goodness of Fit (AGFI), Overall χ^2^, Root Mean Square Error of Approximation (RMSEA), comparative fit index (CFI), and Akaike’s information criterion (AIC) were assessed to provide the validity of how plausibly a specific model fits to the data. Ten iterations of random samples for both EFA and CFA validated that the results were fairly similar through each iteration. Based on each iteration, eigenvalues determined that a three-factor solution for each iteration and goodness of fit measures were consistent. Factor scores were constructed based on summing the individual items with equal weights. A response of strongly disagree equated to 1, disagree to 2, neutral to 3, agree to 4, and strongly agree to 5. For health benefit, a score of 30 was assigned to high benefit, 27–29 to medium benefit, and 26 or less to low benefit. For trustworthiness, a score of 14–15 was assigned to high trust, 12–13 to medium trust, and 11 or less to low trust. For risk concern, a score of 8–10 was assigned to high risk concern, 5–7 to medium risk concern, and 4 or less to low risk concern. General descriptive statistics were performed comparing the three factors using a Pearson Chi-Squared test on categorical variables to assess whether each factor is likely to be independent, and a one-way analysis of variance (ANOVA) test on continuous variables to assess if mean scores between factors are likely to be similar. Multinomial logistic regression analyses were performed, observing the association between demographic variables and each of the three factors. All analyses were performed using SAS (9.4) statistical software packages. A statistically significant association was defined as a two-sided p < 0.05 and a confidence interval of 95%.

## 3. Results

### 3.1. Survey Completion

The survey completion rate in the first wave of the HOLISTIC Cohort Study was 51.0% (556/1090). One participant was not examined due to an incomplete response within the survey (*n* = 555). Among participants, the majority were below age 30 years (74.9%), self-identified as female (79.1%), non-white (51.1%), had been vaccinated with one or more non-COVID-19 vaccines in the last year (81.6%), and had a healthcare provider (70.4%).

### 3.2. Demographics and Vaccine History

Demographics and vaccine history varied significantly across the health sciences colleges (Table 1). For example, health professional students in the college of dentistry and pharmacy were more likely to be non-white than in other colleges. Students in the colleges of dentistry, nursing, and social work were more likely to be age 30 years or older compared to the other colleges. Participants in the college of dentistry, medicine, and pharmacy were more likely to be male. Students in the colleges of medicine or nursing were more likely to have received 2 or more vaccines. Health professional students in the college of nursing were more likely to have a healthcare provider, while students in the college of nursing, public health, and social work were more likely to have seen their provider in the past year. Lastly, participants in the college of dentistry and social work were more likely to not see their doctor because of costs.

### 3.3. COVID-19 Vaccine Hesitancy Domains

**Figure 1:**
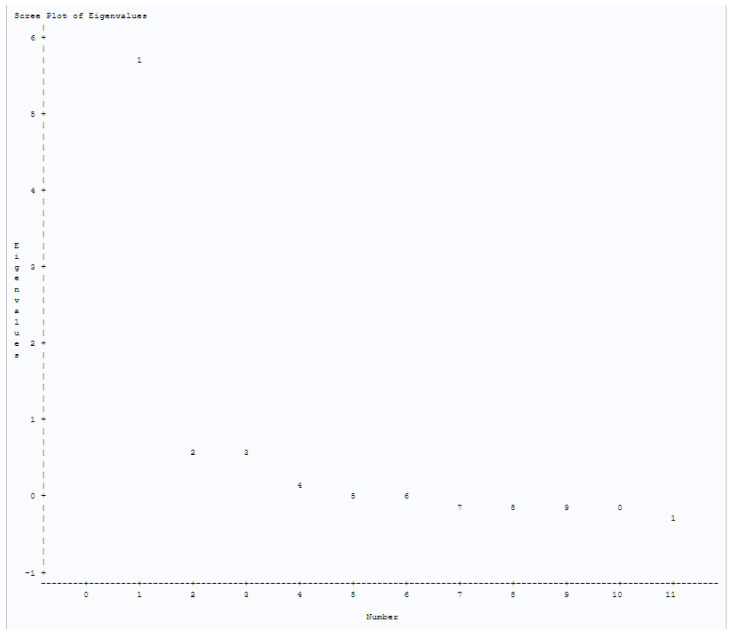
Scree plot of the eigenvalues for the determination of the number of factor groups. The scree plot is used to determine the number of factors to retain in an EFA or principal components to keep in a principal component analysis (PCA). The “elbow” of the scree plot is where the eigenvalues seem to level off and aids in the determination of the number of factors.

**Figure 2:**
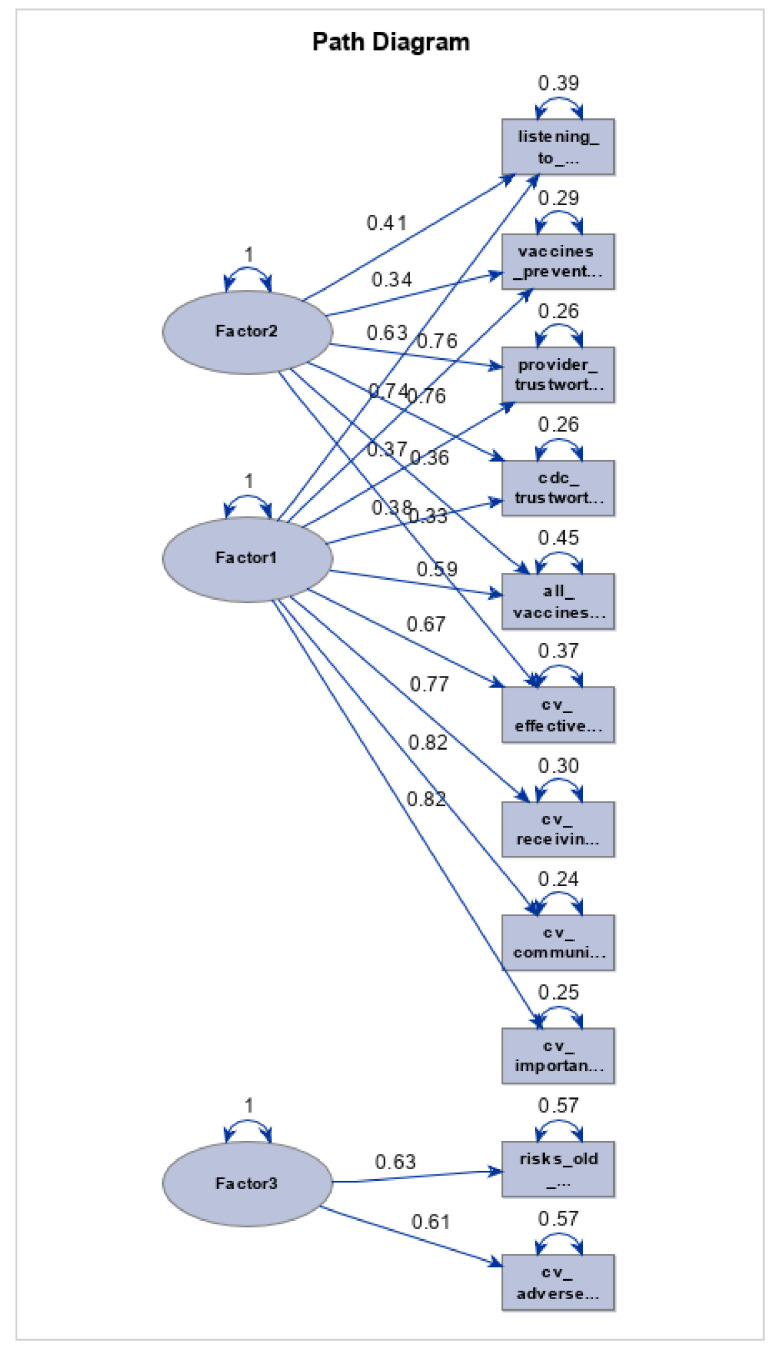
CFA model.

Three domains were identified using the factor analysis, which account for 75.3% of the explained variance (perceived benefit, trustworthiness, and risk) (Table 2). Cronbach’s alpha ranged from 0.64 to 0.922, indicating a moderate to very good internal consistency. A principal component analysis with varimax rotation was conducted on the 11 items assessing COVID-19 vaccine hesitancy (*n* = 277). Aside from eigenvalues, a scree plot confirmed a three-factor solution (Figure 1). For validity of the original factor compared to proposed factors, we performed confirmatory analyses (*n* = 277) to determine the best fitting factor construct (Table 3). Construct 1 was the original factor while proposed constructs (Constructs 2–6) were developed, each having a select number of variables removed. Performance findings reveal that Construct 1 is the best fitting factor construct moving forward, with no substantial differences in fit statistics between constructs (Table 4). Construct 1 Baseline Model Chi-Square and Chi-Square for Absolute Index tests indicate relatively poor model fit (*p* < 0.001) but maintain a high GFI (0.910) and AGFI (0.855), in addition to a fairly low RMSEA value (0.1123), which indicate a relatively adequate fit (Table 4, Figure 2).

### 3.4. COVID-19 Vaccine Hesitancy by Student Demographics, Prior Vaccine Use, and Health Sciences Program

Compared to white, non-Hispanic participants, Hispanic participants perceived a lower likelihood of benefit (OR = 0.43; CI [0.21–0.90], *p* = 0.026) (Table 5). While no significant differences were observed in race/ethnicity in terms of concerns about trustworthiness (Table 6), students who were Asian (OR = 3.10; CI [1.49–6.48], *p* = 0.003) or Hispanic (OR = 2.34; CI [1.01–5.42], *p* = 0.048) reported more concern about risk of taking a COVID-19 vaccine compared to white, non-Hispanics (Table 7). Additionally, health professional students who received ≥2 vaccines in the past year were less likely to have concerns about benefit of a COVID-19 vaccine (OR = 2.50; CI [1.50–4.15], *p* < 0.001) or trustworthiness (OR = 2.71; CI [1.56–4.72], *p* < 0.001) compared to those who had received <2 vaccines. Furthermore, students who had a healthcare provider were less likely to have concerns about risk (OR = 2.04; CI [1.03–4.03], *p* = 0.040) compared to those who did not have a healthcare provider, and health profession students who could not see a provider due to cost demonstrated less trustworthiness (OR = 0.50; CI [0.26–0.97], *p* = 0.041) than students who did not identify cost as a barrier to seeing a healthcare provider in the past 12 months.

Compared to the students in the College of Medicine, students in the Colleges of Applied Health Science (OR = 0.43; CI [0.19–0.96], *p* = 0.040), Pharmacy (OR = 0.38; CI [0.17–0.87], *p* = 0.022), Nursing (OR = 0.35 CI [0.16–0.78], *p* = 0.011), and Social Work (OR = 0.30; CI [0.11–0.78], *p* = 0.014) were significantly less likely to report a potential for benefit (Table 5). Compared to the students in the College of Medicine, students in the College of Applied Health Sciences (OR = 0.39; CI [0.17–0.94], *p* = 0.035), Dentistry (OR = 0.27; CI [0.10–0.76], *p* = 0.013), Nursing (OR = 0.38; CI [0.16–0.94], *p* = 0.037), and Social work (OR = 0.31; [0.11–0.86], *p* = 0.025) were less likely to report trustworthiness (Table 6) and more likely to report concerns about risk (OR 2.80; CI [1.15–6.81], *p* = 0.023 for College of Applied Health Sciences, OR 9.12; CI [2.80–29.75], *p* < 0.001 for Dentistry, OR 3.77; CI [1.47–9.65], *p* = 0.006 for Nursing, OR 3.14; CI [1.02–9.67], *p* = 0.046 for Social Work) (Table 7).

## 4. Discussion

In order to utilize immunity through vaccination as a control measure for the COVID-19 pandemic, adequate vaccination coverage is required, but vaccine hesitancy has made universal uptake of various COVID-19 vaccines challenging. Similar to our study, prior research has also demonstrated an increased likelihood of COVID-19 vaccine uptake amongst those who were previously vaccinated for other infections [38]. Furthermore, we found that individuals in our cohort had less trust in a COVID-19 vaccine if they are not able to afford to see a physician. The inability to access healthcare services may be a driving force for individuals to have less trust in the government and healthcare agencies that are recommending vaccination.

When looking within race and ethnicity, our study shows an increased concern about risk of taking a new vaccine for COVID-19 and its potential side effects amongst Asian, Hispanic, and to a lesser extent African American individuals, rather than a lack of trust or less perceived health benefit. This is consistent with other studies of the general population, which have also demonstrated that much of the vaccine hesitancy in minority populations is due to concerns about vaccine safety [4,5,8,9,39]. Concerns about the risk of potential side effects among these groups has also been shown to not be limited to the general population, but also extends to healthcare workers who identify as a part of these racial and ethnic groups [40]. These findings point to the systemic issues within society that may lead to increased vaccine hesitancy amongst these groups at the general, healthcare worker, and health-professional student levels.

Similar to previous studies in Europe and Africa [41,42], we also found wide variation amongst students in the various health professional colleges and their levels or reasons for being hesitant about a vaccine for COVID-19. Medical students consistently showed to be one of the least hesitant groups across the three factors, along with students in the School of Public Health. On the other hand, applied health students, nursing students, and social work students demonstrated the most hesitancy across all three factors in this study. Furthermore, dentistry students were less likely to trust a COVID-19 vaccine and more likely to have a perceived higher risk of vaccination. Lastly, pharmacy students were found to be trusting and not perceive there to be as much risk in the vaccine but did perceive a lower amount of health benefit from the vaccine. Studies have shown that medical students have a greater prior knowledge of vaccines compared to non-medical students [37], and that pharmacy students overall feel well prepared to address concerns about vaccines with patients [43], which may explain the lower amount of hesitancy seen across the factors in our study. Furthermore, studies have demonstrated increased vaccine hesitancy in individuals with lower awareness levels of vaccines [44,45]. These differences in education on vaccines and potential lack of relevant information seen in previous studies may increase levels of distrust or reluctance of individuals to receive a vaccine in these health science colleges [46]. This underscores the importance of the educational curriculum that is updated consistently to keep up with the various advancements in vaccines. However, prior to looking at the educational curriculum on vaccination, it is important to consider if the differences observed in our study examining students from one university are also present on a larger scale, such as across the state or country.

Other studies that have utilized or modified the WHO SAGE VHS to address hesitancy specifically toward COVID-19, have also completed EFA and CFA to identify different variations of domains from which vaccine hesitancy stems [36,37]. The three factors identified by our EFA accounted for 75.3% of the explained variance. Studies assessing the original VHS found 76% variation [47] and 67% variation [48] utilizing two factors, while another study that modified the VHS to assess hesitancy for COVID-19 found 54% variation utilizing three factors [37]. While each study found differences in variation, the similarity of our findings to these studies suggests that our modified version of the VHS can be used as an appropriate substitute.

The strength of this study is that it compares ethnically, culturally, and socioeconomically diverse students across seven health professional colleges and the factors that drive vaccine hesitancy in each of these populations; research that has never been carried out in the U.S. This provides us the opportunity to begin to understand the factors that influence the willingness of our future health professionals to take up and promote not only the COVID-19 vaccine, but future vaccines that may be created. The limitations of this study include, first, that the study results are based on respondents to a survey in the HOLISTIC Cohort Study at a single U.S. university, and findings in this report may not be representative of all health sciences students in the U.S. As a result, this exposes the study to a selection bias. Second, the participation across health science colleges was not equal with different proportions of students from the various health science colleges participating in the study. Third, vaccine hesitancy was assessed among study participants shortly after the Food and Drug Administration issued an Emergency Use authorization for the first two COVID-19 vaccines in the U.S. (Pfizer-BioNtech COVID-19 Vaccine/BNT162b2 on December 11 2020 [49], and Moderna COVID-19 Vaccine/mRNA-1273 on December 18 2020 [50]), but before the two vaccines have received full FDA approval (August 31 2021 [51] and January 31 2022 [52], respectively). The cross-sectional analysis affects the ability to draw causal relationships, and the patterns of vaccine hesitancy may have changed over time; additional analyses are planned using data to be collected in the second and third waves of enrollment in the HOLISTIC Cohort Study. Fourth, the study results are based on responses to a questionnaire about vaccine hesitancy; additional studies are needed to evaluate the association between self-reported vaccine hesitancy and actual behavior (e.g., vaccination history confirmed by medical records).

## 5. Conclusions

In conclusion, multiple factors influence health professional students’ level of vaccine hesitancy. Those students who have received multiple prior vaccinations are more likely to see health benefit in a COVID-19 vaccine and trust the information received about the vaccine. Students who do not have access to a physician because of cost are less likely to trust information or the agencies that provide information about the COVID-19 vaccine. Health profession students from Asian and Hispanic backgrounds are more likely to have increased concerns of risk surrounding a novel COVID-19 vaccine. Applied health, nursing, social work, and dentistry students were more likely to have higher levels of vaccine hesitancy across the factors studied. Medical, pharmacy, and public health students exhibited some of the lowest hesitancy across the factors of vaccine hesitancy. This provides insight into factors that influence the willingness of our future health professionals to take up and promote not only the COVID-19 vaccine, but also future vaccines that may be created.

## Figures and Tables

**Table 1:** Baseline demographics and vaccine history stratified by health science college.

	All	Applied Health	Dentistry	Medicine	Nursing	Pharmacy	Public Health	Social Work
	(*n* = 555)	(*n* = 93)	(*n* = 45)	(*n* = 133)	(*n* = 101)	(*n* = 85)	(*n* = 48)	(*n* = 50)
**Race/Ethnicity**								
White, Not Hispanic	259 (48.9%)	51 (56.0%)	14 (34.1%)	60 (47.2%)	64 (65.3%)	23 (28.4%)	23 (51.1%)	24 (51.1%)
Asian, Not Hispanic	132 (24.9%)	4 (8.5%)	15 (36.6%)	33 (26.0%)	15 (15.3%)	35 (43.2%)	7 (15.6%)	4 (8.5%)
African American, Not Hispanic	24 (4.5%)	3 (6.4%)	4 (9.8%)	6 (4.7%)	3 (3.1%)	5 (6.2%)	2 (4.4%)	3 (6.4%)
Other Race, Not Hispanic	43 (8.1%)	4 (8.5%)	6 (14.6%)	11 (8.7%)	5 (5.1%)	10 (12.3%)	1 (2.2%)	4 (8.5%)
Hispanic	72 (13.6%)	12 (25.5%)	2 (4.9%)	17 (13.4%)	11 (11.2%)	8 (9.9%)	12 (26.7%)	12 (25.5%)
**Age**								
20–29 years	391 (74.9%)	73 (83.0%)	22 (52.4%)	109 (87.2%)	57 (60.6%)	68 (87.2%)	37 (78.7%)	27 (56.3%)
>=30 years	131 (25.1%)	15 (17.0%)	20 (47.6%)	16 (12.8%)	37 (39.4%)	10 (12.8%)	10 (21.3%)	21 (43.7%)
**Gender**								
Male	111 (20%)	17 (18.3%)	32 (72.7%)	45 (33.8%)	7 (6.9%)	26 (30.6%)	3 (6.3%)	2 (4.0%)
Female	438 (79.1%)	76 (81.7%)	11 (25.0%)	86 (64.7%)	93 (92.1%)	58 (68.2%)	45 (93.7%)	48 (96.0%)
Other Response	5 (0.9%)	0 (0.0%)	1 (2.3%)	2 (1.5%)	1 (1.0%)	1 (1.2%)	0 (0.0%)	0 (0.0%
**Prior Vaccine History (any vaccine)**								
<= 2 vaccines	242 (43.7%)	42 (45.2%)	23 (51.1%)	40 (30.3%)	37 (36.6%)	40 (47.1%)	29 (60.4%)	31 (62.0%)
>2 vaccines	312 (56.3%)	51 (54.8%)	22 (48.9%)	92 (69.7%)	64 (63.4%)	45 (52.9%)	19 (39.6%)	19 (38.0%)
**Healthcare Provider**								
Yes	380 (70.4%)	64 (70.3%)	32 (74.4%)	91 (70.5%)	78 (80.4%)	52 (63.4%)	33 (68.8%)	30 (60.0%)
No	160 (29.6%)	27 (29.7%)	11 (25.6%)	38 (29.5%)	17 (19.6%)	30 (36.6%)	15 (31.2%)	20 (40.0%)
**Could Not See Doctor Because of Costs**								
Yes	90 (16.8%)	12 (13.0%)	10 (23.3%)	19 (15.2%)	19 (19.2%)	10 (12.3%)	9 (18.7%)	11 (22.5%)
No	447 (83.2%)	80 (87.0%)	33 (76.7%)	106 (84.8%)	80 (80.8%)	71 (87.7%)	39 (81.3%)	38 (77.5%)
**Last Visit to Doctor for Routine Checkup**								
<1 year ago	329 (61.0%)	55 (61.8%)	24 (54.5%)	72 (57.6%)	65 (65.0%)	49 (58.3%)	32 (68.1%)	32 (64.0%)
>= 1 year ago	210 (39.0%)	34 (38.2%)	20 (45.5%)	57 (42.4%)	35 (35.0%)	35 (41.7%)	15 (31.9%)	18 (36.0%)

**Table 2:** Factor analysis and reliability of COVID-19 vaccine hesitancy survey questions.

Factors	Factor Score	Reliability
**Factor 1: Benefit**		
Being vaccinated for COVID-19 or coronavirus would be important for the health of others in my community	0.83	0.92
A vaccine developed for COVID-19 or coronavirus would be important for my health	0.83	
I would receive a vaccine developed for COVID-19 or coronavirus	0.71	
Getting vaccines is a good way to protect me from disease	0.69	
A vaccine developed for COVID-19 or coronavirus would be effective	0.65	
All vaccines offered by the government program in my community are beneficial	0.53	
**Factor 2: Trustworthy**		
The information I receive about vaccines from my doctor or healthcare provider is reliable and trustworthy	0.78	0.87
The information I receive about vaccines from public health officials is reliable and trustworthy	0.73	
Generally, I do what my doctor or healthcare provider recommends about vaccines	0.56	
**Factor 3: Risk Concern**		
I would be concerned about serious adverse effects of a vaccine developed for COVID-19 or coronavirus	0.59	0.64
New vaccines carry more risks than older vaccines	0.58	

Factor analysis shows a breakdown of individual items grouped into a group of factors. We use Cronbach’s alpha of reliability to measure internal consistency, or how closely a related set of items are as a group. Higher reliability signifies that items are strongly associated with each other.

**Table 3:** List of different confirmatory factor analysis constructs.

Construct	Variables Removed	# of Variables Removed	Total Variables
** 1 **	None (original model with three factors)	0	11
** 2 **	Removal of question: **getting vaccines is a good way to protect me from disease** from Factor 1	1	10
** 3 **	Removal of question: **generally, I do what my doctor or healthcare provider recommends about vaccines** from Factor 2	1	10
** 4 **	Variables from Models 2 + 3	2	9
** 5 **	Removal of questions: **I would be concerned about serious adverse effects of a vaccine developed for COVID-19 or coronavirus and new vaccines carry more risks than older vaccines** from Factor 3	2	9
** 6 **	Variables from Models 4 + 5	4	7

List of the original construct (10 items in 3 factors) in comparison to different variations of constructs (e.g., dropping individual items from specific factors).

**Table 4:** Performance of different confirmatory factor analysis constructs.

Fit Summary	Construct 1	Construct 2	Construct 3	Construct 4	Construct 5	Construct 6
** Baseline Model Chi-Square (*p*-value) ^a^**	<0.001	<0.001	<0.001	<0.001	<0.001	<0.001
** Chi-Square for Absolute Index (*p*-value) ^a^**	<0.001	<0.001	<0.001	<0.001	<0.001	<0.001
** GFI ^b^**	0.9100	0.9259	0.9464	0.9654	0.9000	0.9658
** AGFI ^b^**	0.8552	0.8726	0.9079	0.9351	0.8269	0.9263
** RMSEA ^c^**	0.1123	0.1113	0.0850	0.0750	0.1374	0.0908

Performance results of original and additional constructs using several CFA performance tests. **^a^** If both the Baseline Model Chi-Square estimate and the Chi-Square for the Absolute Index are less than 0.05, this will indicate that the a priori model may not be the best fit for the vaccine hesitancy survey questions; ^b^ The Goodness of Fit Index (GFI) and the Adjusted GFI (AGFI) are interpreted in that an estimate closer to 1 indicates a better fitting model; ^c^ Root Mean Square Error of Approximation (RMSEA) assesses how far a hypothesized model is from a perfect model. Interpretation of this statistic is that the closer the RMSEA is to 0, the better the model fit.

**Table 5:** Multinomial logistic regression on health benefit scoring outcome.

	High vs. Low Health Benefit		Medium vs. Low Health Benefit	
Variables	OR (CI)	*p*-value	OR (CI)	*p*-Value
**Race/Ethnicity** (ref = White, Not Hispanic)				
White, Not Hispanic	-	-	-	-
Asian, Not Hispanic	1.18 (0.62–2.28)	0.613	1.22 (0.60–2.45)	0.585
African American, Not Hispanic	0.37 (0.13–1.41)	0.585	0.42 (0.13–1.41)	0.161
Other Races, Not Hispanic	0.84 (0.35–2.03)	0.694	0.91 (0.35–2.34)	0.841
Hispanic	0.43 (0.21–0.90)	**0.026**	0.63 (0.30–1.34)	0.232
**Prior Vaccine History** (ref = ‘ <= 2 vaccines’)				
<= 2 vaccines	-	-	-	-
> 2 vaccines	2.50 (1.50–4.15)	**<0.001**	1.66 (0.97–2.85)	0.067
**Age** (ref = ‘20–29’)				
20–29 years	-	-	-	-
>=30 years	1.10 (0.61–1.99)	0.755	0.86 (0.45–1.63)	0.634
**Health Science College** (ref = ’Medicine’)				
Medicine	-	-	-	-
Applied Health Science	0.43 (0.19–0.96)	**0.040**	0.78 (0.32–1.87)	0.574
Dentistry	0.42 (0.15–1.17)	0.098	0.81 (0.27–2.40)	0.699
Nursing	0.35 (0.16–0.78)	**0.011**	0.54 (0.22–1.31)	0.172
Pharmacy	0.38 (0.17–0.87)	**0.022**	0.56 (0.22–1.40)	0.213
Public Health	1.30 (0.40–4.24)	0.659	2.55 (0.76–8.62)	0.132
Social Work	0.30 (0.11–0.78)	**0.014**	0.72 (0.27–1.96)	0.523
**Healthcare Provider** (ref = ‘No’)				
No	-	-	-	-
Yes	0.69 (0.39–1.22)	0.202	0.86 (0.46–1.61)	0.633
**Could Not See Doctor Because of Costs** (ref = ‘No’)				
No	-	-	-	-
Yes	0.87 (0.45–1.66)	0.662	0.85 (0.42–1.70)	0.638
**Last Visit to Doctor for Routine Checkup** (ref = ‘<= 1 year ago’)				
≤1 year ago	-	-	-	-
>1 year ago	0.83 (0.48–1.42)	0.499	0.76 (0.43–1.36)	0.358

If OR > 1; the odds of increased health benefit, relative to Low Health Benefit, is XXX times more likely for (sub-category X) compared to (reference category). Example: the odds of Higher Health Benefit, relative to Low Health Benefit, are 2.50 times more likely for someone who has had more than 2 vaccines, compared to someone who has had <=2 vaccines. If OR < 1; the odds of an increased health benefit, relative to Low Health Benefit, is XXX times less likely for (sub-category X) compared to (reference category). Example: the odds of Higher Health Benefit, relative to Low Health Benefit is 0.43 times less likely for Hispanics compared to Non-Hispanic White participants.

**Table 6:** Multinomial logistic regression on trustworthy scoring outcome.

	High vs. Low Trustworthy		Medium vs. Low Trustworthy	
Variables	OR (CI)	*p*-Value	OR (CI)	*p*-Value
**Race/Ethnicity** (ref = White, Not Hispanic)				
White, Not Hispanic	-	-	-	-
Asian, Not Hispanic	0.91 (0.45–1.82)	0.785	1.30 (0.62–2.71)	0.483
African American, Not Hispanic	0.54 (0.17–1.73)	0.297	0.89 (0.26–3.02)	0.845
Other Races, Not Hispanic	0.74 (0.28–1.93)	0.534	1.23 (0.46–3.32)	0.681
Hispanic	0.64 (0.28–1.47)	0.292	1.35 (0.59–3.09)	0.472
**Prior Vaccine History** (ref = ‘<= 2 vaccines’)				
<=2 vaccines	-	-	-	-
>2 vaccines	2.71 (1.56–4.72)	**<0.001**	2.55 (1.43–4.56)	**0.002**
**Age Group** (ref = ‘20–29′)				
20–29	-	-	-	-
>=30	1.13 (0.60–2.14)	0.697	0.97 (0.50–1.89)	0.927
**Health Science College** (ref = ‘Medicine’)				
Medicine	-	-	-	-
Applied Health Science	0.39 (0.17–0.94)	**0.035**	0.70 (0.28–1.78)	0.457
Dentistry	0.27 (0.10–0.76)	**0.013**	0.40 (0.13–1.24)	0.112
Nursing	0.38 (0.16–0.94)	**0.037**	0.89 (0.35–2.30)	0.810
Pharmacy	0.71 (0.27–1.87)	0.493	0.99 (0.36–2.74)	0.980
Public Health	1.25 (0.38–4.11)	0.714	1.38 (0.39–4.94)	0.621
Social Work	0.31 (0.11–0.86)	**0.025**	0.75 (0.26–2.13)	0.457
**Healthcare Provider** (ref = ‘No’)				
No	-	-	-	-
Yes	1.22 (0.66–2.25)	0.534	1.02 (0.53–1.93)	0.961
**Could Not See Doctor Because of Costs** (ref = ‘No’)				
No	-	-	-	-
Yes	0.50 (0.26–0.97)	**0.041**	0.52 (0.26–1.04)	0.065
**Last Visit to Doctor for Routine Checkup** (ref = ‘<= 1 year ago’)				
≤1 year ago	-	-	-	-
>1 year ago	1.10 (0.61–1.98)	0.747	1.00 (0.54–1.85)	0.996

If OR > 1; the odds of Higher Trust, relative to Low Trust, is XXX times more likely for (sub-category X) compared to (reference category). Example: the odds of Higher Trust, relative to Low Trust, are 2.71 times more likely for someone who has had more than 2 vaccines, compared to someone who has had <=2 vaccines. If OR < 1; the odds of Higher Trust, relative to Low Trust, is XXX times less likely for (sub-category X) compared to (reference category). Example: the odds of Higher Trust, relative to Low Trust is 0.39 times less likely for students in the College of Applied Health Sciences compared to students in the College of Medicine.

**Table 7:** Multinomial logistic regression on risk concern scoring outcome.

	High vs. Low Risk Concern		Medium vs. Low Risk Concern	
Variables	OR (CI)	*p*-Value	OR (CI)	*p*-Value
**Race/Ethnicity** (ref = White, Not Hispanic)				
White, Not Hispanic	-	-	-	-
Asian, Not Hispanic	3.10 (1.49–6.48)	**0.003**	2.45 (1.37–4.40)	**0.003**
African American, Not Hispanic	2.78 (0.76–10.13)	0.121	1.49 (0.50–4.44)	0.472
Other Races, Not Hispanic	1.35 (0.49–3.71)	0.556	1.15 (0.53–2.49)	0.719
Hispanic	2.34 (1.01–5.42)	**0.048**	1.39 (0.70–2.73)	0.345
**Prior Vaccine History** (ref = ‘<= 2 vaccines’)				
<=2 vaccines	-	-	-	-
>2 vaccines	0.69 (0.39–1.23)	0.209	0.87 (0.55–1.36)	0.535
**Age Group** (ref = ‘20–29’)				
20–29	-	-	-	-
>=30	0.66 (0.33–1.34)	0.249	0.82 (0.48–1.40)	0.470
**Health Science College** (ref = ‘Medicine’)				
Medicine	-	-	-	-
Applied Health Science	2.80 (1.15–6.81)	**0.023**	1.49 (0.77–2.89)	0.234
Dentistry	9.12 (2.80–29.75)	**<0.001**	2.82 (0.99–8.03)	0.052
Nursing	3.77 (1.47–9.65)	**0.006**	2.71 (1.35–5.42)	**0.005**
Pharmacy	2.06 (0.80–5.33)	0.136	1.53 (0.77–3.05)	0.227
Public Health	1.10 (0.32–3.77)	0.879	1.49 (0.68–3.30)	0.323
Social Work	3.14 (1.02–9.67)	**0.046**	2.41 (1.04–5.57)	**0.040**
**Healthcare Provider** (ref = ‘No’)				
No	-	-	-	-
Yes	2.04 (1.03–4.03)	**0.040**	1.29 (0.79–2.11)	0.305
**Could Not See Doctor Because of Costs** (ref = ‘No’)				
No	-	-	-	-
Yes	1.67 (0.80–3.45)	0.170	0.95 (0.52–1.75)	0.865
**Last Visit to Doctor for Routine Checkup** (ref = ‘<= 1 year ago’)				
≤1 year ago	-	-	-	-
>1 year ago	1.24 (0.66–2.30)	0.503	1.25 (0.78–2.02)	0.358

If OR > 1; the odds of higher concern towards the risk of the Vaccine, relative to Lower Concern, is XXX times more likely for (sub-category X) compared to (reference category). Example: the odds of having higher Risk Concern, relative to Lower Concern, is 2.34 times more likely for Hispanic participants compared to Non-Hispanic White participants.

## Data Availability

Wave 1 of the HOLISTIC Cohort Study data underlying results presented in the study is available in the Indigo database, a public data repository hosted by University of Illinois Chicago (doi: 10.25417/uic.19641330).

## Supplementary Materials

The following supporting information can be downloaded at: https://www.mdpi.com/article/10.3390/vaccines10091566/s1, Table S1: Select BRFSS Survey Questions and Table S2: WHO SAGE VHS Question Alterations.

## References

[1] Randall T., Sam C., Tartar A., Murray P., Cannon C. More Than 11.9 Billion Shots Given: COVID-19 Tracker. Bloomberg. https://www.bloomberg.com/graphics/covid-vaccine-tracker-global-distribution/.

[2] World Health Organization. https://www.who.int/emergencies/diseases/novel-coronavirus-2019/covid-19-vaccines.

[3] Burke P.F., Masters D., Massey G. (2021). Enablers and barriers to COVID-19 vaccine uptake: An international study of perceptions and intentions. Vaccine.

[4] Neumann-Böhme S., Varghese N.E., Sabat I., Barros P.P., Brouwer W., Van Exel J., Schreyögg J., Stargardt T. (2020). Once we have it, will we use it? A European survey on willingness to be vaccinated against COVID-19. Eur. J. Health Econ..

[5] Wang J., Jing R., Lai X., Zhang H., Lyu Y., Knoll M.D., Fang H. (2020). Acceptance of COVID-19 Vaccination during the COVID-19 Pandemic in China. Vaccines.

[6] Callaghan T., Moghtaderi A., Lueck J.A., Hotez P., Strych U., Dor A., Fowler E.F., Motta M. (2021). Correlates and disparities of intention to vaccinate against COVID-19. Soc. Sci. Med..

[7] Larson H.J., De Figueiredo A., Xiahong Z., Schulz W.S., Verger P., Johnston I.G., Cook A.R., Jones N.S. (2016). The State of Vaccine Confidence 2016: Global Insights Through a 67-Country Survey. EBioMedicine.

[8] Prickett K.C., Habibi H., Carr P.A. (2021). COVID-19 Vaccine Hesitancy and Acceptance in a Cohort of Diverse New Zealanders. Lancet Reg. Health West. Pac..

[9] Albahri A.H., Alnaqbi S.A., Alshaali A.O., Shahdoor S.M. (2021). COVID-19 Vaccine Acceptance in a Sample from the United Arab Emirates General Adult Population: A Cross-Sectional Survey, 2020. Front. Public Health.

[10] Jamison A.M., Quinn S.C., Freimuth V.S. (2018). “You don’t trust a government vaccine”: Narratives of institutional trust and influenza vaccination among African American and white adults. Soc. Sci. Med..

[11] Reuben R., Aitken D., Freedman J.L., Einstein G. (2020). Mistrust of the medical profession and higher disgust sensitivity predict parental vaccine hesitancy. PLoS ONE.

[12] Allen J.D., Feng W., Corlin L., Porteny T., Acevedo A., Schildkraut D., King E., Ladin K., Fu Q., Stopka T.J. (2021). Why are some people reluctant to be vaccinated for COVID-19? A cross-sectional survey among U.S. Adults in May–June 2020. Prev. Med. Rep..

[13] Fu C., Wei Z., Pei S., Li S., Sun X., Liu P. (2020). Acceptance and preference for COVID-19 vaccination in health-care workers: A Comparative Analysis and Discrete Choice Experiment. MedRxiv.

[14] Udow-Phillips M., Lantz P.M. (2020). Trust in Public Health Is Essential Amid the COVID-19 Pandemic. J. Hosp. Med..

[15] Biswas N., Mustapha T., Khubchandani J., Price J.H. (2021). The Nature and Extent of COVID-19 Vaccination Hesitancy in Healthcare Workers. J. Community Health.

[16] Karafillakis E., Dinca I., Apfel F., Cecconi S., Wűrz A., Takacs J., Suk J., Celentano L.P., Kramarz P., Larson H.J. (2016). Vaccine hesitancy among healthcare workers in Europe: A qualitative study. Vaccine.

[17] Roy B., Kumar V., Venkatesh A. (2020). Health Care Workers’ Reluctance to Take the Covid-19 Vaccine: A Consumer-Marketing Approach to Identifying and Overcoming Hesitancy. NEJM Catalyst Innovations in Care Delivery.

[18] Courage K.H. It’s Essential to Understand Why Some Health Care Workers Are Putting off Vaccination. Vox. https://www.vox.com/22214210/covid-vaccine-health-care-workers-safety-fears.

[19] Mustapha T., Khubchandani J., Biswas N. (2021). COVID-19 Vaccination Hesitancy in Students and Trainees of Healthcare Professions: A Global Assessment and Call for Action. Brain Behav. Immun. Health.

[20] Kelekar A.K., Lucia V.C., Afonso N.M., Mascarenhas A.K. (2021). COVID-19 vaccine acceptance and hesitancy among dental and medical students. J. Am. Dent. Assoc..

[21] Lucia V.C., Kelekar A., Afonso N.M. (2020). COVID-19 vaccine hesitancy among medical students. J. Public Health.

[22] Manning M.L., Gerolamo A.M., Marino M.A., Hanson-Zalot M.E., Pogorzelska-Maziarz M. (2021). COVID-19 vaccination readiness among nurse faculty and student nurses. Nurs. Outlook.

[23] Mascarenhas A.K., Lucia V.C., Kelekar A., Afonso N.M. (2021). Dental students’ attitudes and hesitancy toward COVID-19 vaccine. J. Dent. Educ..

[24] Larson H.J., Jarrett C., Eckersberger E., Smith D.M.D., Paterson P. (2014). Understanding Vaccine Hesitancy around Vaccines and Vaccination from a Global Perspective: A Systematic Review of Published Literature, 2007–2012. Vaccine.

[25] WHO Euro Working Group on Vaccine Communications, Istanbul, Turkey, 13–14 October 2011.

[26] World Health Organization Regional Office for Europe The Guide for Tailoring Immunization Programs. Increasing Coverage of Infant and Child Vaccination in the WHO European Region. https://www.euro.who.int/__data/assets/pdf_file/0003/187347/The-Guide-to-Tailoring-Immunization-Programmes-TIP.pdf.

[27] MacDonald N.E., The SAGE Working Group on Vaccine Hesitancy (2015). Vaccine Hesitancy: Definition, Scope and Determinants. Vaccine.

[28] Akther T., Nur T. (2022). A model of factors influencing COVID-19 vaccine acceptance: A synthesis of the theory of reasoned action, conspiracy theory belief, awareness, perceived usefulness, and perceived ease of use. PLoS ONE.

[29] Betsch C., Schmid P., Heinemeier D., Korn L., Holtmann C., Böhm R. (2018). Beyond confidence: Development of a measure assessing the 5C psychological antecedents of vaccination. PLoS ONE.

[30] Thomson A., Robinson K., Vallée-Tourangeau G. (2016). The 5As: A practical taxonomy for the determinants of vaccine uptake. Vaccine.

[31] U.S. Food and Drug Administration FDA. https://www.fda.gov/emergency-preparedness-and-response/coronavirus-disease-2019-covid-19/covid-19-vaccines.

[32] Dommaraju S.R., Rivera S.G., Rocha E.G., Bicknell S., Loizzo D., Mohammad A., Rajan P., Seballos A., Datta A., Ahmed R. (2022). Health professional students at the University of Illinois Chicago (HOLISTIC) Cohort Study: A protocol. PLoS ONE.

[33] Minority-Serving Institution Status. University of Illinois at Chicago. https://chancellor.uic.edu/minority-serving-designations/.

[34] Centers for Disease Control and Prevention (CDC) (2019). Behavioral Risk Factor Surveillance System Survey Questionnaire. U.S. Department of Health and Human Services, Centers for Disease Control and Prevention. https://www.cdc.gov/brfss/questionnaires/pdf-ques/2019-BRFSS-Questionnaire-508.pdf.

[35] (2014). Report of the SAGE Working Group on Vaccine Hesitancy. World Health Organization. https://www.asset-scienceinsociety.eu/sites/default/files/sage_working_group_revised_report_vaccine_hesitancy.pdf.

[36] Shen X., Dong H., Feng J., Jiang H., Dowling R., Lu Z., Lv C., Gan Y. (2021). Assessing the COVID-19 vaccine hesitancy in the Chinese adults using a generalized vaccine hesitancy survey instrument. Hum. Vaccines Immunother..

[37] Sadaqat W., Habib S., Tauseef A., Akhtar S., Hayat M., Shujaat S.A., Mahmood A. (2021). Determination of COVID-19 Vaccine Hesitancy Among University Students. Cureus.

[38] Goffe L., Antonopoulou V., Meyer C.J., Graham F., Tang M.Y., Lecouturier J., Grimani A., Bambra C., Kelly M.P., Sniehotta F.F. (2021). Factors associated with vaccine intention in adults living in England who either did not want or had not yet decided to be vaccinated against COVID-19. Hum. Vaccines Immunother..

[39] Chen J.-H., Shiu C.-S. (2022). Race, ethnicity and COVID-19 vaccine concerns: A latent class analysis of data during early phase of vaccination. SSM Popul. Health.

[40] Swann M.C., Bendetson J., Johnson A., Jatta M., Schleupner C.J., Baffoe-Bonnie A. (2022). Examining Drivers of COVID-19 Vaccine Hesitancy Among Healthcare Workers. Infect. Control Hosp. Epidemiol..

[41] Gautier S., Luyt D., Davido B., Herr M., Cardot T., Rousseau A., Annane D., Delarocque-Astagneau E., Josseran L. (2022). Cross-sectional study on COVID-19 vaccine hesitancy and determinants in healthcare students: Interdisciplinary trainings on vaccination are needed. BMC Med. Educ..

[42] Yendewa S.A., Ghazzawi M., James P.B., Smith M., Massaquoi S.P., Babawo L.S., Deen G.F., Russell J.B.W., Samai M., Sahr F. (2022). COVID-19 Vaccine Hesitancy among Healthcare Workers and Trainees in Freetown, Sierra Leone: A Cross-Sectional Study. Vaccines.

[43] Wick J.A., Henneman A. (2021). Pharmacy student perceptions of their preparedness to address vaccine hesitancy and refusal. Curr. Pharm. Teach. Learn..

[44] Robertson E., Reeve K.S., Niedzwiedz C.L., Moore J., Blake M., Green M., Katikireddi S.V., Benzeval M.J. (2021). Predictors of COVID-19 vaccine hesitancy in the UK household longitudinal study. Brain, Behav. Immun..

[45] Saied S.M., Saied E.M., Kabbash I.A., Abdo S.A.E. (2021). Vaccine hesitancy: Beliefs and barriers associated with COVID-19 vaccination among Egyptian medical students. J. Med Virol..

[46] Sandler K., Srivastava T., Fawole O.A., Fasano C., Feemster K.A. (2019). Understanding vaccine knowledge, attitudes, and decision-making through college student interviews. J. Am. Coll. Health.

[47] Domek G.J., O’Leary S.T., Bull S., Bronsert M., Contreras-Roldan I.L., Ventura G.A.B., Kempe A., Asturias E.J. (2018). Measuring vaccine hesitancy: Field testing the WHO SAGE Working Group on Vaccine Hesitancy survey tool in Guatemala. Vaccine.

[48] Shapiro G.K., Tatar O., Dube E., Amsel R., Knauper B., Naz A., Perez S., Rosberger Z. (2018). The vaccine hesitancy scale: Psychometric properties and validation. Vaccine.

[49] U.S. Food and Drug Administration. https://www.fda.gov/news-events/press-announcements/fda-takes-key-action-fight-against-covid-19-issuing-emergency-use-authorization-first-covid.

[50] U.S. Food and Drug Administration. https://www.fda.gov/news-events/press-announcements/fda-takes-additional-action-fight-against-covid-19-issuing-emergency-use-authorization-second-covid.

[51] U.S. Food and Drug Administration. https://www.fda.gov/news-events/press-announcements/fda-approves-first-covid-19-vaccine#:~:text=Since%20Dec.

[52] U.S. Food and Drug Administration. https://www.fda.gov/news-events/press-announcements/coronavirus-covid-19-update-fda-takes-key-action-approving-second-covid-19-vaccine.

