# Supplementary material for: COVID-19 Vaccine Hesitancy among Health Professional Students: Cross-Sectional Data from the First Wave of the HOLISTIC Cohort Study
Source: Vaccines (Basel). 2022 Sep 19;10(9):1566. doi: 10.3390/vaccines10091566 (PMC9502320; doi:10.3390/vaccines10091566)
Supplement: Supplementary file 1 [file vaccines-10-01566-s001.zip › vaccines-1903883-supplementary.pdf]

**Table S1** Select BRFSS Survey Questions

| Question | Potential Responses |
|---|---|
| Do you have one person you think of as your personal doctor or health care provider? | 1. Yes, only one<br>2. Yes, more than one<br>3. No<br>4. I do not know<br>5. Prefer not to answer |
| Was there a time in the past 12 months when you needed to see a doctor but could not because of cost? | 1. Yes<br>2. No<br>3. I do not know<br>4. Prefer not to answer |
| About how long has it been since you last visited a doctor for a routine checkup? | 1. Anytime less than or equal to one year ago<br>2. More than 1 year but less than or equal to 2 years ago<br>3. More than 2 years but less than 5 years ago<br>4. Five or more years ago<br>5. I do not know<br>6. Never<br>7. Prefer not to answer<br>8. |
| What was your sex at birth? Was it male or female? | 1. Male<br>2. Female<br>3. I do not know<br>4. Prefer not to answer |
| What is your date of birth? | _ _/_ _/_ _ _ _ |
| Are you Hispanic, Latino/a, or of Spanish origin? | 1. Mexican, Mexican American, Chicano/a<br>2. Puerto Rican<br>3. Cuban<br>4. Another Hispanic, Latino/a, or Spanish origin<br>5. No<br>6. I do not know<br>7. Prefer not to answer |
| Which one of these groups would you say *best* represents your race? *(Select only one)* | 1. White<br>2. Black or African American<br>3. American Indian or Alaska Native<br>4. Asian<br>a. Asian Indian<br>b. Chinese<br>c. Filipino<br>d. Japanese<br>e. Korean<br>f. Vietnamese<br>g. Other Asian<br>5. Pacific Islander<br>a. Native Hawaiian<br>b. Guamanian or Chamorro |

| | |
|---|---|
| | c. Samoan<br>d. Other Pacific Islander<br>6. Middle Eastern or North African<br>a. Lebanese<br>b. Iranian<br>c. Egyptian<br>d. Syrian<br>e. Moroccan<br>f. Algerian<br>g. Other Middle Eastern or North African<br>7. Other<br>8. None of the above<br>9. I do not know<br>10. Prefer not to answer |
| During the past 12 months, have you had either a flu vaccine that was sprayed in your nose or a flu shot injected into your arm? | 1. Yes<br>2. No<br>3. I do not know<br>4. Prefer not to answer |
| *[If A to #83]* During what month and year did you receive your most recent flu vaccine that was sprayed in your nose or flu shot injected into your arm? | 1. _ _ / _ _ _ _<br>2. I do not know<br>3. Prefer not to answer |
| Have you received a tetanus shot in the past 10 years? | 1. Yes, received Tdap<br>2. Yes, received tetanus shot, but not Tdap<br>3. Yes, received tetanus shot but not sure what type<br>4. No, did not receive any tetanus shot in the past 10 years<br>5. I do not know<br>6. Prefer not to answer |
| Have you ever had a pneumonia shot also known as a pneumococcal vaccine? | 1. Yes<br>2. No<br>3. I do not know<br>4. Prefer not to answer |
| Have you ever had the Human Papilloma virus vaccination or HPV vaccination? | 1. Yes<br>2. No<br>3. I do not know<br>4. Prefer not to answer |
| *[If A to #87]* How many HPV shots did you receive? | 1. _ _ Number of shots<br>2. All shots<br>3. I do not know<br>4. Prefer not to answer |
| Do you consider yourself to be transgender? | 1. Yes, transgender, male to female<br>2. Yes, transgender, female to male<br>3. Yes, transgender, gender nonconforming<br>4. No<br>5. I do not know<br>6. Prefer not to answer |

This table contains the subset of questions from the modified BRFSS survey administered to participants in the HOLISTIC study [25] that were used in this study.

**Table S2** WHO SAGE VHS Question Alterations

| Original WHO SAGE VHS Questions | Modified WHO SAGE VHS Questions Used |
|---|---|
| Childhood vaccines important for my child's health. | **A vaccine developed for COVID-19 or coronavirus would be** important for my health. |
| Childhood vaccines are effective. | **A vaccine developed for COVID-19 or coronavirus would be** effective. |
| Having my child vaccinated is important for the health of others in my community. | **Being vaccinated for COVID-19 or coronavirus would be** important for the health of others in my community. |
| All childhood vaccines offered by the government program in my community are beneficial. | All vaccines offered by the government program in my community are beneficial. |
| New vaccines carry more risks than older vaccines. | New vaccines carry more risks than older vaccines. |
| The information I receive about vaccines from the vaccine program is reliable and trustworthy. | The information I receive about vaccines from **public health officials** is reliable and trustworthy. |
| | The information I receive about vaccines from **my doctor or healthcare provider** is reliable and trustworthy. |
| Getting vaccines is a good way to protect my child/children from disease. | Getting vaccines is a good way to protect **me** from disease. |
| Generally, I do what my doctor or health care provider recommends about vaccines for my child/children. | Generally, I do what my doctor or health care provider recommends about vaccines. |
| I am concerned about serious adverse effects of vaccines. | I **would be** concerned about serious adverse effects of **a vaccine developed for COVID-19 or coronavirus.** |

The underlined portion of each question from the original WHO SAGE VHS Questions indicates the words that were changed or omitted in the questionnaire used in this study. The **bolded** portion of the question in the modified WHO SAGE VHS Questions used in this study indicates the words that were added in place of the underlined portion from the original question.
